# Combining non-invasive methods for the rapid assessment of mammalian richness in a transect-quadrat survey scheme – Case Study of the Horsh Ehden Nature Reserve, North Lebanon

**DOI:** 10.3897/zookeys.119.1040

**Published:** 2011-07-15

**Authors:** Manal R. Nader, Shadi El Indary, Bachir Abi Salloum, Manale Abou Dagher

**Affiliations:** 1 Director, Institute of the Environment, University of Balamand, Lebanon; Assistant Professor, Faculty of Science; 2 Instructor, Institute of the Environment, University of Balamand, Lebanon; 3 Post-Doc fellow, Department of Pediatrics and Endocrinology, University of Michigan, Ann Arbor, USA; 4 Instructor, Institute of the Environment, University of Balamand, Lebanon

**Keywords:** Mammals, diversity, Horsh Ehden Nature Reserve, Lebanon

## Abstract

Lebanon lacks updated information related to the status of mammalian species, their richness and distribution. This study aimed at developing a rapid assessment methodology combining three non-invasive techniques based on the transect-quadrat survey scheme to measure mammalian richness at the Horsh Ehden Nature Reserve. The achieved results showed that the combination of the three techniques, droppings, footprints and photo-trapping for the rapid assessment of mammalian richness supported by Geographical Information System applications is highly effective. Twenty visits covering twenty quadrats out of 49 over a period of nine months allowed the detection of 12 of the 14 targeted species with droppings providing the majority of evidence while footprints and photo-trapping being of equal efficiency. The method can be easily replicated in any region to rapidly assess mammalian richness and the area of activity of the detected species and therefore directing conservation and management activities towards species of interest.

## Introduction

Existing baseline data on biodiversity in the Middle East region is poor and sporadic, varies from one country to another and from one species to another. Biodiversity assessments, species status and population dynamic publications are insufficient, particularly for mammalian species (Tohmé et al. 1985, Darwish et al. 1997, Bunaian et al. 1998, Bunaian et al. 2001, Abu Baker et al. 2002, Abu Baker et al. 2003, Yigit et al. 2003, Emre Can 2004, Qarqaz et al. 2005, Abd Rabou et al. 2007, Temple et al. 2009).

Mammalian presence is identified through various non-invasive techniques, mainly footprints, droppings, and feeding signs (Cavallini 1994, Sadlier et al. 2003, Price et al. 2005, Gompper et al. 2006, Abd Rabou et al. 2007, Abi-Said et al. 2007, Bonnaud et al. 2007, De Marinis et al. 2009). Nevertheless, traditional non-invasive techniques to assess mammalian richness are quite time consuming and require extensive resource investment. Lately, new surveying techniques for mammalian studies have become widespread including the increasing dependence on Motion Sensor Cameras (Foresman 2000, Grogan and Lindzey 1999, Trolle and Kery 2005). In addition, mammalian conservation and management strategies are dependent on the efficient and reliable methods for rapid assessment of species richness and abundance (Grogan and Lindzey 1999, Silveira et al. 2003, Trolle and Kery 2005, Gompper et al. 2006), especially in regions where knowledge of mammalian richness is poor and where human and material resources are limited.

The objective of this study was to develop and test a methodology that combines quadrat-transect survey schemes along with non-invasive methods for the rapid assessment of mammalian richness using the Horsh Ehden Nature Reserve (HENR), North Lebanon as the study area.

## Materials and Methods

### Study area

The HENR is situated 100 Km north of the capital Beirut (34°19'N, 36°00' ) and occupies a surface area of approximately 1,775 hectares. It ranges in altitude from 1,300 m to 1,950 m with slopes between 10% and 80%. The mean temperature is 9.3°C and the annual rainfall average is 1060 mm with 95 days of snowfall on more than 50% of the protected area. The habitat is a combination of open Mediterranean shrub land, pine/oak forest and cedar/fir forest. Until present, approximately 1058 species of plants, mammals, invertebrates and birds have been recorded in HENR, among them 26 species of mammals (MOE/UL/UNDP 2004). Fourteen out of the 26 are considered as medium to large sized mammals and were targeted in this study.

### Sampling

**Figure 1.**
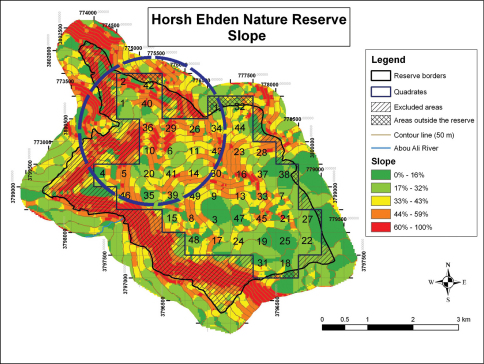
Quadrats and gradient slopes; Blue circle indicates the forested area.

The methodology was based on Williams et al. 2002 with modifications. An ortho-rectified four band satellite image of the HENR dated from the year 2007 was purchased, while several maps showing layers which include the borders of the Reserve, limits of the buffer zone, contour lines (50m) and the land use map (2002) were obtained from the Lebanese Ministry of Environment (http://www.moe.gov.lb). A map of the HENR using a Geographical Information System (GIS) application was produced illustrating the slope gradients (Fig. 1) that allowed identification of the accessible and non-accessible areas for surveying. The accessible area of the Reserve was then divided into 49 quadrats, 500 × 500 m each, giving a total surface area of 250,000 m2 per quadrat (Fig. 1).

**Figure 2.**
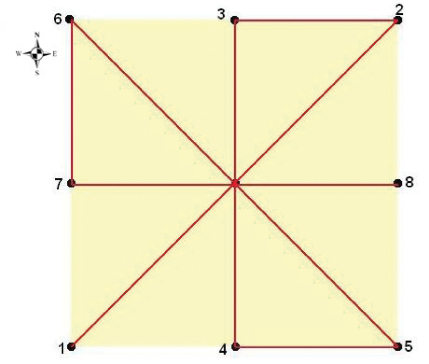
Quadrat travel route.

In each quadrat, a line transect sampling was adopted which involves the observation of individuals and their activity along a specific tract (Williams et al. 2002). In total 12,656 m2 surface area in each quadrat was surveyed where observations covered 3,164 m on foot with a 4 m sight range (2 m to the right and 2 m to the left of the surveyor; Fig. 2). Each quadrat was surveyed over a period of one day.

Animal footprints and droppings spotted during surveying were directly measured, photographed, and identified when possible, and coordinates recorded on an E-trex Summit Global Positioning System (GPS) device (Garmin Ltd. and its subsidiaries, Olathe, Kansas, USA). Photographs were further analyzed and the corresponding species identified according to the following four references: Animal Tracks and Signs (Bang 2004), Tracks and Trailcraft (Jaeger 2002), Mammals of North Africa and the Middle East (Stuart 2008) and Mammals of the Holy Land (Qumsiyeh 1996).

**Figure 3.**
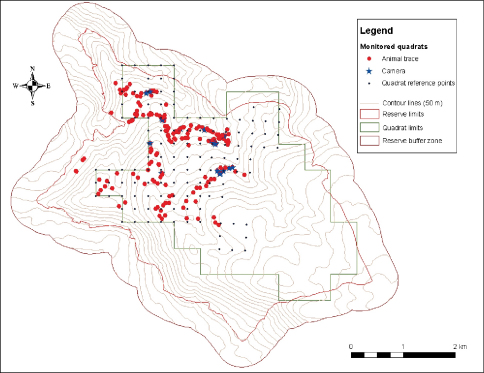
Mammalian distribution map showing the location of mammalian activity and the location of the Motion Sensor Cameras.

Recorded points were then plotted on the map (Fig. 3) using the MapSource 5.4 software (Garmin Ltd. and its subsidiaries, Olathe, Kansas, USA) to classify zones with high mammalian activity. After localizing mammalian activity, two motion sensor Cuddeback Capture Cameras (Non typical Inc., Falls Park, Wisconsin, USA) with set bait to attract wildlife were deployed to allow visual identification of mammalian species and to further confirm their presence in the Reserve. Photos were extracted on a weekly basis in the field and cameras relocated to predefined new locations. Coordinates of each photo-trapped animal were then downloaded on the mammalian distribution map (Fig. 3).

The testing of the sampling methodology was carried-out in the forested area of the Reserve covering 20 quadrats and their surrounding ecotones (Fig. 1). This area was targeted due to its accessibility during the snowy winter season (the open areas are at high altitudes and not accessible in the winter season), the cover provided to mammals to avoid human disturbance, in addition to the fact that anthropogenic activities increase exponentially during the spring and summer seasons in the open areas and their surrounding (grazing, camping, and hiking). Field activities started on December 1, 2008 and ended on August 31, 2009 with one quadrat surveyed per visit.

## Results

In total, 253,120 m2 were surveyed in 20 days over a period of nine months (one quadrat per day) where 12 medium to large size mammals were identified (Table 1). Two hundred and twelve points were recorded for animal droppings and footprints including 17 points where photos were taken (Fig. 3). Coordinates for the location where photographs of mammals were not captured were excluded from the distribution map. Of the 12 identified medium to large sized mammals, five were detected only by droppings (42%); four by droppings, footprints and photos (33%); one by droppings and footprints (8%); one by droppings and photos (8%); and one by footprints and photos (8%) (Table 1). In addition, droppings were collected for 11 of the 12 species (92%), footprints for six species (50%) and photos for six species (50%).

## Discussion

The combination of three non-invasive methods allowed a rapid and extensive coverage of a large surface area of the Reserve that culminated in the identification of 12 medium and large sized mammal species out of 14 listed in previous reports in a relatively short period of time. The use of GIS permitted the exclusion of inaccessible regions from the on-set of sampling activities that led to a substantial reduction in the effort that needed to be invested to cover the target area. In addition, knowledge of the gradient slopes and the main geographical obstacles that may be encountered in each quadrat beforehand allowed the complete sampling of one quadrat per day.

The transect-line sampling revealed that droppings are the most common identification technique for mammalian richness in the HENR (92%), while footprints and photos accounted for 50% each. Droppings tend to remain in the natural environment for longer periods of time than footprints, while photo-trapping is highly dependent on the animal passing in front of the Motion Sensor Cameras. Nevertheless, one mammal, the Persian Squirrel, was identified only through footprints in the snow and its presence in the Reserve was further confirmed through several photos. In addition, during the dry season that stretches for more than five months and the scarce water sources at the HENR lead to the ground drying up for long periods making mammalian footprints very difficult to find. The footprints of the six species detected at the HENR through this technique were found either in snow and/or mud during the wet season.

Even though transect-line sampling tended to be the most efficient in determining mammalian richness, several factors need to be taken into consideration, more specifically for footprints in addition to the challenges experienced from adverse weather conditions. For example, snow conditions may only be appropriate in certain areas for footprint identification and in some cases only for a few days. Furthermore, dense canopy produces thick plant litter that does not allow the mammal to leave footprint markings. Therefore, in addition to the team being qualified in the identification of local fauna, it should run on a flexible schedule that will allow it to take advantage of fresh snowfall and rain. Consequently, assessment of mammalian richness based on the transect-quadrat survey scheme should concentrate on the collection of droppings while still investing effort in detecting footprints where the habitat is suitable. Fresh snow and wet clay and humid soils after rainfall and around water sources will increase the probability of finding mammalian footprints and as a result increase the chances of detecting animals that may not be detected by other techniques.

Photo-trapping was used to confirm the presence of mammals detected by the transect-quadrat survey by installing the cameras in areas where mammalian activity was detected. It was anticipated that the majority of the identified mammals will be photo-trapped. But contrary to expectations and even though bait was installed, only six out of the 12 targeted species were recorded using this technique. This could be due to the fact that the cameras were installed for only one week in the selected spots reducing the chances of the animal passing in front of the sensor. Nevertheless, the extracted photographs revealed that all the photo-trapped species were active at night, a period where the team could not carry-out any sampling activities. In addition, they provided evidence of adults of several species accompanied by juveniles revealing the presence of breeding populations at the HENR. This information is proving to be essential in directing future research towards species considered of value at the national and regional scales. In addition to its low disturbance impact, the installation and functioning of Motion Sensor Cameras requires low investment and low team experience making it quite attractive in regions where human and material resources limit mammalian richness research activities.

The achieved results showed that the combination of the three non-invasive techniques for the rapid assessment of mammalian richness (droppings, footprints and photo-trapping) supported by GIS application is of outmost relevance. Twelve of the 14 targeted species were identified in a relatively short period of time and their area of activity plotted on a map therefore demonstrating the effectiveness of the approach at the HENR. With the widespread availability of GIS applications, this approach can be easily replicated in any region around the world to rapidly assess mammalian richness and the area of activity of the detected species and therefore direct conservation and management activities towards species of interest.

**Table 1.** List of recorded mammals at the HENR and techniques of their identification.

*Scientific Name*	*Common Name*	*Identification Technique*		
		*Dropping*	*Footprint*	*Photo*
*Sus scrofa* Linnaeus, 1758	Wild Boar	x	x	x
*Vulpes vulpes* (Linnaeus, 1758)	Red Fox	x	x	x
*Felis silvestris* Schreber, 1777	Common Wild Cat	x	x	x
*Martes foina* (Erxleben, 1777)	Beech Marten	x	x	x
*Canis aureus* Linnaeus, 1758	Golden Jackal	x	x	
*Mustela nivalis* Linnaeus, 1766	Least Weasel	x		
*Hyaena hyaena* (Linnaeus, 1758)	Barbary Hyaena	x		
*Erinaceus concolor* Martin, 1838	East European Hedgehog	x		
*Hystrix indica* Kerr, 1792	Indian Crested Porcupine	x		
*Lepus capensis* Linnaeus, 1758	Cape Hare	x		
*Meles meles* (Linnaeus, 1758)	Eurasian Badger	x		x
*Sciurus anomalus* Gueldenstaedt, 1785	Persian Squirrel		x	x
